# The efficacy of fish oil supplements in the treatment of depression: food for thought

**DOI:** 10.1038/tp.2016.243

**Published:** 2016-12-06

**Authors:** J A Bastiaansen, M R Munafò, K M Appleton, A J Oldehinkel

**Affiliations:** 1 Department of Psychiatry, Interdisciplinary Center Psychopathology and Emotion Regulation, University of Groningen, University Medical Center Groningen, Groningen, The Netherlands; 2 Department of Education and Research, Friesland Mental Health Care Services, Leeuwarden, The Netherlands; 3 MRC Integrative Epidemiology Unit at the University of Bristol, Bristol, UK; 4 UK Centre for Tobacco and Alcohol Studies, School of Experimental Psychology, University of Bristol, Bristol, UK; 5 Department of Psychology, Faculty of Science and Technology, Bournemouth University, Poole, UK

A recent meta-analysis and meta-regression of 13 randomized clinical trials by Mocking *et al.*^1^ concluded that supplementation with omega-3 fatty acids, found naturally in fatty fish, has a beneficial effect in patients with major depressive disorder (MDD), especially for higher doses of the eicosapentaenoic acid (EPA) and in patients taking antidepressants. Novel treatments for MDD are certainly desired. However, in our view the evidence in this study does not solve the academic debate on the efficacy of omega-3 fatty acids for MDD. Some food for thought.

## Meta-analysis: not more than the sum of its parts

On the basis of the widely accepted GRADE system, a recent Cochrane review evaluated the overall quality of the evidence of studies on omega-3 fatty acids and depressive symptomatology (*n*=26) as very low,^2^ and the body of evidence as composed of a limited number of predominantly small studies at high risk of selection, performance or attrition bias. Poor evidence quality downgrades the credibility of overall effect size estimates, particularly when the evidence for an effect appears to be driven by poorer quality studies.

Study selection concerns aside, Mocking *et al.*^1^ found no association between study effect size and study quality as operationalized by the 5-point Jadad score in their subset of 13 studies. Jadad scores, however, simply indicate whether a study reports a double-blind randomized trial and reports drop-outs and withdrawals, resulting in a maximal score for 9 of the 13 reviewed studies. This minimal variation largely reduced the power to detect associations with study effect size. More importantly, the Jadad score ignores highly relevant aspects such as risk of bias and study precision (1/s.e.). Analyses conducted on studies with low risk of bias have consistently produced nonsignificant effect estimates.^2^ Moreover, based on the mean standardized differences and s.e.'s reported in their Figure 1, we found that the studies included in Mocking *et al.*^1^ show an inverse association between study effect size and study precision (*r*=−0.344): less precise trials produced larger effect sizes. To illustrate the impact that less precise studies can have on meta-analytic results, we repeated the meta-analysis (based on the data provided in Mocking *et al.*^1^) but without the least precise study^3^ (*N*=20), which reduced the overall effect size from a standardized mean difference (SMD) of 0.398 (95% confidence interval (CI): 0.114, 0.681, *P*=0.006) to 0.317 (95% CI: 0.051, 0.582, *P*=0.019). In addition, excluding the second-least precise study^4^ (*N*=22) further reduces the effect size to 0.227 (95% CIs: 0.001, 0.453, *P*=0.049). Thus, the observed effect of omega-3 fatty-acid supplementation on depression seems largely driven by the most imprecise studies.

## Meta-regression: the more trials the merrier

**Figure 1:**
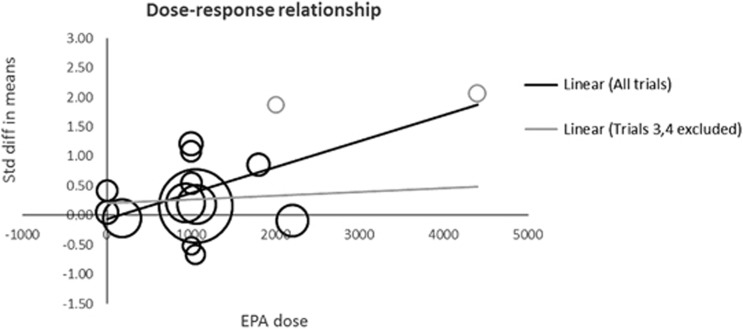
Dose–response relationship. Circles represent the effect size of the individual trials scaled by their sample size. The gray circles represent the studies by Nemets *et al.*^3^ and Su *et al.*^4^ (top right), which have the smallest sample sizes and the largest effect sizes. The dose–response relationship is depicted as a solid line for the linear trend based on all trials (*r*=0.6) and a gray line discarding Nemets *et al.*^3^ and Su *et al.*^4^ (*r*=0.1). EPA, eicosapentaenoic acid.

Based on nine univariate meta-regressions (one for each study characteristic) across 13 trials, Mocking *et al.*^1^ concluded that omega-3 fatty-acid supplementation in MDD patients is especially beneficial in patients using antidepressants and for higher doses of EPA. A low number of trials reduces the probability of a true-negative finding. Whereas the number of trials here may not be exceptionally low compared with other meta-regressions, detecting moderator effects requires more powerful analyses than are employed in most published studies.^5^ Especially when high heterogeneity is present across studies, as is the case in Mocking *et al.*^1^ (*I*^2^=73%, *t*^2^=0.171), power of 80% to detect even the largest of the modest moderator effects reported in Mocking *et al.*^1^ may not be achieved except with a much larger number of trials.^5^ Perhaps counterintuitively, low statistical power also decreases the probability that an observed effect that reaches nominal statistical significance actually reflects a true effect.^6, 7^ The risk of false-positive findings is further increased by the substantial number of statistical tests conducted in this study.^7, 8^ Indeed, neither of the results (antidepressants, *P*=0.044; EPA dose, *P*=0.009) survives correction for multiple comparisons (Bonferroni *P*-value=0.05/9 =0.006), and the EPA dose–response relationship is mainly attributable to the two least precise studies^3, 4^ (Figure 1).

## Meta-regression: correlation is not causation

The conclusions on EPA dose and antidepressant use were not based on randomization of these characteristics. Meta-regression is observational and, therefore, susceptible to confounding; it does not allow causal inference.^8^ Hence, the associations found with EPA dose and antidepressant use could be due to other, known or unknown, trial characteristics. That findings from this meta-regression do not necessarily align with results from intervention studies is illustrated by the largest clinical trial available to date (*N*=432),^9^ which stratified randomization by antidepressant use and found evidence for neither an interaction between treatment group and antidepressant use, nor benefit from EPA supplementation among the subgroup of patients also taking antidepressants (*n*=174).

Meta-analyses are critical to evidence-based medicine, but may lead to biased conclusions if the quality of available evidence is not adequately considered. Findings from meta-regression should be interpreted with particular caution, especially when suggesting clinical implications. Even if unbiased, a statistically significant result is not necessarily clinically relevant, and one may wonder whether, for instance, a decrease of 0.04 on the 17-item Hamilton Depression Rating Scale with every 100 mg increase in EPA dose is meaningful. In our view, the current evidence supporting the use of omega-3 fatty-acid supplementation in depression remains weak and clinical implications should be tempered.
